# Volumetric MRI of dorsal root ganglia as a biomarker for disease progression and response to AAV treatment in a mouse model of Fabry disease

**DOI:** 10.1371/journal.pone.0334840

**Published:** 2025-10-24

**Authors:** Fuqiang Zhao, Shipeng Yuan, Charalambos Kaittanis, Mugdha Deshpande, Abirami Kugadas, Katayoun Derakhchan, Wanida Ruangsiriluk, Rizwana Islam, Natalia Boukharov, Paul McQuade, Johannes Tauscher, Christopher T. Winkelmann, Talakad G. Lohith

**Affiliations:** Takeda Development Center America, Inc., Cambridge, Massachusetts, United States of America; International University of Health and Welfare, School of Medicine, JAPAN

## Abstract

Noninvasive and objective biomarkers for disease-associated pathology are critical for clinical trials. For Fabry disease, one important pathological change due to the deficiency of the lysosomal enzyme α-galactosidase A (α-GAL) caused is accumulation of globotriaosylceramide (Gb3) in dorsal root ganglion (DRG) neurons, which manifests as the overall DRG hypertrophy. Magnetic resonance imaging (MRI) has been successfully used to noninvasively measure DRG enlargement in Fabry patients, and DRG volumetric MRI can be a potential noninvasive biomarker for Gb3 accumulations in DRG neurons in clinical trials. To evaluate disease progression and treatment response in preclinical proof-of-concept studies, we developed an *in vivo* MRI method to measure DRG size in the G3Stg/GLA knockout mouse model of Fabry disease. Compared to the wild type mice, the DRG enlargement in the Fabry mice was observed as early as 8 weeks of age, and a single administration of the human α-GAL-encoding adeno-associated virus (AAV_GLA_) normalized the enlarged DRG to the age-matched wild type mice. The DRG normalization was observed within 4 weeks of gene therapy (12 weeks of age) and was sustained up to 24 weeks of age. Furthermore, behavioral testing and histological/immunohistochemistry analyses of the DRG tissues corroborated the MRI findings. Volumetric DRG MRI has the sensitivity to measure Gb3 pathology-induced DRG volume changes and treatment response in live mice and can be a translational imaging biomarker in clinical trials for Fabry disease.

## Introduction

Fabry disease (FD) is an inherited, lysosomal storage disorder caused by the deficiency of the lysosomal enzyme α-galactosidase A (α-GAL) and presents with painful neuropathy as one of the earliest clinical presentations [1]. The function of α-GAL is to catabolize glycosphingolipids with terminal α-galactosyl groups. With impaired α-GAL function, intracellular glycosphingolipids, predominantly globotriaosylceramide (Gb3), accumulate in cells of different organs including in the dorsal root ganglion (DRG) neurons [2–4]. The accumulation of intracellular Gb3 can cause cell vacuolation and enlargement [5], including DRG hypertrophy. In addition to overall DRG hypertrophy, Gb3 accumulation may also cause DRG neuron destruction which is presumed to be the cause of neuropathic pain syndrome in Fabry patients [6]. A study in a Fabry mouse model suggested that Gb3 accumulation in DRG impairs neuronal integrity and ion channel function, causing pain and small fiber pathology in Fabry disease [4]. Since DRG enlargement and neuropathic pain in Fabry patients are more likely from the same pathological source, DRG volume can potentially be used as a substitute biomarker for the neuropathic pain in Fabry patients.

Magnetic resonance imaging (MRI) has been used to measure DRG volume in humans, and DRG volumetric MRI have demonstrated that DRG volumetric changes are associated with peripheral neuropathic diseases such as Fabry disease [7–13], diabetic neuropathy [14], neurofibromatosis type 1 and 2 [15,16], and polyradiculoneuropathy [17]. Non-invasive, longitudinal MRI measurement of DRG volume may be used as a biomarker to trace the pathological progression in DRG of these diseases and potentially evaluate the efficacy of disease-modifying therapies. In drug development settings, MRI measurement of DRG volume in animals can be very useful but requires technical development and critical assessment for translational purposes. MRI measurement of DRG volume has been reported in larger animal species such as dogs [18], non-human primates (NHPs) [19], and post-mortem rats [20]. However, there are no reports about MRI measurement of DRG volume in mice. Since mice are commonly used in drug discovery and preclinical evaluation, there is a need to develop an MRI method to measure DRG volume in mice.

The α-GAL knockout (GLAko) and symptomatic G3Stg/GLAko (GLAko mice with human Gb3 synthase overexpression) mouse models of Fabry disease show prominent lysosomal inclusions in DRGs among other tissues [21,22]. Recent reports have shown the excised mouse DRGs from both mouse models of Fabry disease are enlarged with marked accumulation of Gb3 and are associated with the poor outcomes of peripheral sensory system [23]. These Fabry mouse models can be used to evaluate the feasibility/sensitivity of volumetric DRG MRI methods as a translational biomarker to measure the treatment effect, such as in Fabry gene therapy (GT) in the preclinical development stage.

In previous volumetric DRG MRI studies in humans, MRI protocols, including T1-weighted, T2-weighted, proton-density-weighted, and T2*-weighted sequences, have been developed to measure DRG volume [14,17,24,25]. However, the most widely used MRI protocol is the T2-weighted fast spin-echo sequence with short-tau-inversion-recovery (STIR) [7]. STIR is used to suppress the signal intensity of peri-ganglion fat which would confound DRG segmentation if not sufficiently suppressed.

In this study, we used an MRI protocol with T2-weighted fast spin echo sequence to measure the DRG volume *in vivo* in mice. To evaluate the reliability of this MRI method, a test-retest study with wild-type mice was performed. The feasibility and sensitivity of this imaging method as a biomarker for disease-induced DRG volume change was evaluated in Fabry mice with and without GT treatment. Furthermore, histological analysis of collected DRG tissue and behavioral tests from Fabry and wild-type mice were performed to support imaging findings.

## Materials and methods

### Animal preparation

Animal welfare complied with all applicable regulations, and all *in vivo* procedures described in this study were approved by the Institutional Animal Care and Use Committees of Takeda Pharmaceuticals Inc. and Northeastern University. Throughout the study period, any mice exhibiting signs of distress were immediately euthanized in accordance with humane guidelines. Euthanasia was performed using 5% isoflurane. For test-retest repeatability study, five male C57BL/6 adult mice from Taconic (Boston, MA, USA) were used. For the gene therapy study with Fabry mice, five male C57BL/6 wild type mice and thirteen male G3Stg/GLAko Fabry mice [26] also from Taconic were used. G3Stg/GLAko Fabry mice were generated by cross-breeding GLAko Fabry mice with transgenic mice expressing human Gb3 synthase [26]. This advanced model accumulates more than 10-fold of Gb3 substrates in tissues compared to age-matched GLAko mice and pathologically progresses significantly with age similar to humans [26], and therefore is more optimal model to evaluate the volumetric DRG MRI method.

For MRI scanning, animals were anesthetized using 5% isoflurane for induction and with ~2% isoflurane for maintenance. During MRI data acquisition, body temperature was monitored and maintained by a rectal probe and air heater (35–37 °C), and respiration was monitored by a balloon secured to the mouse’s chest and a pressure transducer (SA Instruments, Inc., Stony Brook, NY USA). Isoflurane level was adjusted to maintain the respiration rate in the range of approximately 60–120 breaths/minute during MRI data acquisition.

### MRI studies

**Fig 1:**
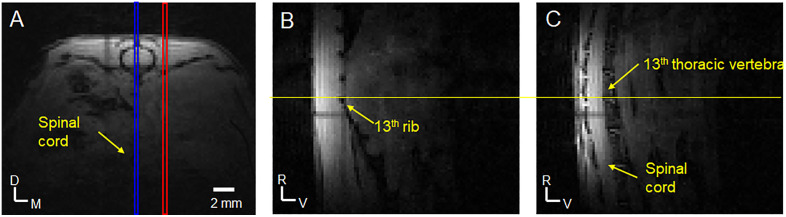
Anatomical images to identify vertebral levels. Based on the axial image (A), two sagittal images (B & C) were acquired as indicated by red box and blue box in (A). Image (B) was used to identify the position of 13^th^ rib (the lowest circular region with hypo-intensity in the caudal direction). From this anatomical marker, the 13^th^ thoracic vertebra is identified (C). DRG MRI data in axial slices were acquired around the 13^th^ thoracic vertebra level. D: dorsal; V: ventral; M: medial; R: rostral.

All MRI measurements were performed on a 7T, 20-cm bore Bruker Biospec system (Bruker, Billerica, MA, United States). The gradient coil was a 12-cm inner diameter set with a maximum gradient strength of 0.2 T/m and a rise time of 120 µs. An actively decoupled 10-mm diameter single channel/single loop surface coil positioned beneath the lumbar spinal cord of the mouse was used as the radiofrequency (RF) receiver, while an actively decoupled 72-mm diameter volume coil was used as the RF transmitter. The surface coil was fixed on the Bruker rat cradle. The mouse was carefully secured in a small home-made holder in supine position with its dorsum lying on the surface coil. The holder part between mouse and coil was open to let the spinal cord be close to the surface coil. Anatomical images in three directions (axial, sagittal, and coronal) were acquired by Bruker Tri-pilot protocol (FLASH sequence) with the following parameters: slice thickness 0.5 mm; field of view (FOV) 2.56 × 2.56 cm^2^; matrix size 128 × 128; in-plane spatial resolution 0.2 × 0.2 mm^2^; repetition time (TR) 50 ms; echo time (TE) 2.4 ms; flip angle 10°; number of averages 4; total acquisition time 26 sec. From the coronal image, the alignment of the spinal cord with the magnet direction can be assessed. The animal position could be easily adjusted by moving the small animal holder to align the spinal cord in the coronal direction with the magnet z-direction. After animal positioning, radiofrequency power was calibrated, and magnetic field homogeneity was optimized by automatic global shimming. Fig 1A shows the axial image from a Tri-pilot scan. From this axial image, two sagittal slices with the positions indicated in Fig 1A were acquired and shown in Figs 1B and 1C. In the sagittal image from the slice adjacent to the spinal column, the 13^th^ rib was identified and used as an anatomical marker to locate the appropriate vertebral levels. The spinal cord segment corresponding to thoracic vertebra 13 was positioned in the iso-center of the magnet. This vertebra level corresponds to the lumbar 4 (L4) segment of spinal cord (see Fig 7 in [27]).

Rapid acquisition with relaxation enhancement (RARE) protocol with 3D mode was used to acquire DRG MRI data in an axial segment centering in the L4 segment of spinal cord. The observed DRG in this spinal cord segment is L4 DRG. To minimize fat contribution to MRI images, the spectral fat saturation was used to suppress fat signal. RARE parameters: TR = 2000 msec, effective TE = 42 msec, field of view (FOV) = 2 cm (left right direction) × 1.5 cm (dorsal-ventral direction) × 0.7 cm (rostral-caudal direction), matrix = 256 × 192 × 24, resolution = 78 × 78 × 292 μm^3^, RARE factor = 20, number of averages = 8, acquisition time = 1 hour 2 min.

### Study design and vector administration

Recombinant AAV_GLA_ and AAV_null_ were prepared and characterized using established internal procedures (Takeda), as described previously [28]. The viral vectors AAV_GLA_, which encoded human α-GAL GLA, and AAV_null_, which lacked any transgenic sequence, were formulated in phosphate buffered saline with 0.001% Pluronic F-68 with the concentration of 1.25 × 10^12^ vg/ml. The test and control articles were intravenously injected at 6.25 × 10^12^ vg/kg (Table 1) in a volume dose of 5 ml/kg to 8-week-old G3Stg/GLAko Fabry mice (Table 1). Baseline MRI data was acquired at 8-week of age, before viral vector injections. The subsequent MRI data were acquired at the ages of week 12, week 16, week 20, and week 24. Following the last MRI data acquisition at the age of week 24, the animals were euthanized with 5% isoflurane, and the spinal column tissues were collected for immunohistochemistry and Hematoxylin and eosin (H&E) staining analysis.

**Table 1:** Group designation and treatment protocol.

Group	Mouse (male)	N	Gene Therapy	AAV Dose
Wild-type	C57BL/6	5	None	N/A
Fabry	G3Stg/GLAko	3	None	N/A
Fabry + AAV_null_	G3Stg/GLAko	5	AAV9-null (rAAV9-MY011)	6.25 × 10^12^ vg/kg
Fabry + AAV_GLA_	G3Stg/GLAko	5	AAV9-GLA (rAAV9-MD022)	6.25 × 10^12^ vg/kg

### MRI image analysis

**Fig 2:**
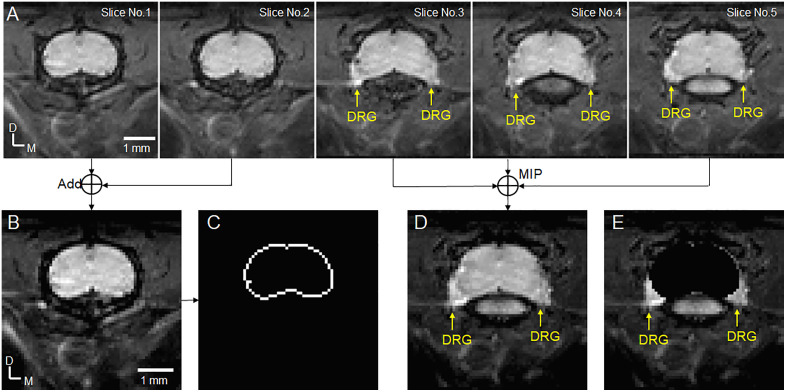
DRG delineation. (A) images from 5 consecutive axial slices (slice thickness = 0.29 mm). The DRGs are observed in slices No. 3, 4 and 5, but not in slices No. 1 and 2. (B) MRI image of spinal cord without DRGs by averaging slice No. 1 and 2. (C) Spinal cord edge from the spinal cord image in (B). (D) MRI image of the spinal cord with DRGs from maximum intensity projection (MIP) of slice No. 3, 4 and 5. (E) DRG image from (D) by zeroing the spinal cord region defined in (C). D: dorsal, M: medial.

MRI data was analyzed using NIH ImageJ (version: 1.53K) together with custom MATLAB routines (Mathworks, Version 7.12.0, Natick, MA). The MRI images from the axial slices were observed on an image-by-image basis (Fig 2A), and the images with DRG were identified (slice No.3, 4, 5). There is no clear contrast between the DRG and spinal cord. As demonstrated in S1 Fig, the size and shape of spinal cord in these slices with the DRG are similar with the spinal cord in the adjacent slices (slice No.1 and 2). The morphological information of spinal cord from the two adjacent slices at the proximal side of DRG was used to delineate the DRG from spinal cord as follows:

1) To align the spinal cord in these slices to the same location by adjusting images in the dorsal-ventral direction if needed (i.e., the acquired axial images may not be perfectly perpendicular to the spinal cord, a small dorsal–ventral shift based on the spinal cord outline can be applied to achieve alignment)
2) The MRI images from the two spinal cord slices were averaged (Fig 2B)
3) MATLAB “edge” function was used to detect the spinal cord edge (Fig 2C)
4) The maximum intensity projection (MIP) was performed on the slices with DRG (Slice No. 3, 4 and 5) to get the image as shown in Fig 2D
5) The pixels within the spinal cord edge were zeroed to get DRG image (Fig 2E).

The DRG was manually delineated from other surrounding tissues based on the higher signal intensity of the DRG (S2B and S3B Figs). The delineated pixels were added together as the cross-sectional area (CSA), which is the maximal CSA in the axial direction of DRG, to represent the DRG volume. The DRG segmentation method assumes that the size and shape of the spinal cord remain consistent across continuous axial slices within a small segment. The spinal cord images from two adjacent slices on the proximal side of the L4 DRG were used for spinal cord delineation. The spinal cord images from the distal side of the L4 DRG were not included. The reason for this exclusion is that if the slices are too far apart, the assumption of consistency in shape and size of spinal cord becomes less valid. As shown in Fig 2, the L4 DRG spans approximately 0.9 mm in the longitudinal direction, meaning the distance between the proximal and distal spinal cord images is slightly greater than 1 mm. When averaging spinal cord images over such a distance, the assumption of uniformity may not hold as expected, which could result in blurred boundaries in the averaged spinal cord image. This blurring effect may introduce errors or variability in spinal cord delineation. The Matlab “Edge” function, which detects the spinal cord boundaries by calculating image gradients, is sensitive to such blurring. A less distinct gradient can make edge detection less robust, potentially compromising the accuracy of the spinal cord segmentation.

### MRI data analysis

For test-retest data analysis, a scatterplot was created to visually relate the DRG CSA measurements from the test MRI scan and retest MRI scan. Intraclass correlation coefficient (ICC, one-way random effects) was calculated as a reliability measurement [29] by the MATLAB code downloaded from MATLAB Central File Exchange [30]. A Bland–Altman plot [31] was used to observe variance in the DRG CSA measurements, and limits of agreement (LoA) were calculated from the mean differences and the standard deviation of the differences [32].

Statistical analyses were performed using Microsoft Excel’s t-test function (Microsoft Corporation, Redmond, WA). To compare age-related DRG CSA and body weight changes in Fabry mice, data from the Fabry group and the Fabry + AAV_null_ group were pooled together to compare with the DRG CSA data from the wildtype group and assessed using Student’s t-test with a closed testing procedure. The comparison was performed at the endpoint at which the maximum difference was predicted. If the resulting two-sided p-value was < 0.05, the same comparison was repeated retrospectively to determine the earliest time point with a significant difference. To detect the AAV_GLA_ treatment effect on the DRG CSA, the data from the Fabry + AAV_GLA_ group was compared with the pooled data from the Fabry group and the Fabry + AAV_null_ group and the wildtype group by two-sided Student’s t-test.

### Histological evaluation

The spinal cord columns with DRG tissues and foot pads were quickly dissected from wild-type and Fabry mice at ages of 8 and 24 weeks, the samples were rinsed with cold phosphate buffered saline (PBS), then fixed in 10% neutral buffered formalin (NBF) (Thermo Fisher Scientific, Catalog No. SF100−20) shaking on an orbital shaker at room temperature for 48 h before being washed and transferred to PBS. The samples containing bone tissues were subjected to decalcification with RapidCal Immuno Decalcifier (BBC Biochemical, Mt Vernon, WA, USA, Catalog No. 6089) following fixation on an orbital shaker at room temperature for another 24 h. Then, the decalcified samples were rinsed in running RODI (reverse osmosis deionized) water for 1–2 h. All fixed samples were processed for paraffin-embedded blocks by a Tissue TEK VIP Tissue processor (SAKURA Fine Tek USA, Inc., Torrance, CA, USA) and sectioned at 5μm. The tissue sections were collected for immunohistochemistry (IHC) assay and performed on an automated Leica Bond system (Leica Biosystems, Deer Park, IL, USA) using a Bond Polymer Refine Detection kit (DS9800, Leica Biosystems). Antibodies against the following targets were obtained as indicated: LAMP1 (Abcam, Waltham, MA, USA, Catalog No. ab24170), Myelin Protein Zero (MPZ, Abcam, Catalog No. ab183868). The stained sections were also counterstained with hematoxylin. The stained slides were dehydrated, mounted with Cytoseal*60 Mounting Medium (Thermo Fisher Scientific), and covered with coverslips by a Leica CV5030 Fully Automated Glass Cover slipper (Leica Biosystems). Hematoxylin and eosin (H&E) staining was conducted by an Automated Leica ST5020CV5030 Stainer Integrated Workstation (Leica Biosystems). All stained slides were scanned with a Leica AT2 Scanner (Leica Biosystems). Aperio ImageScope and HALO^®^ image system (Indica Labs, Albuquerque, NM, USA) were reviewed. Representative images were selected for figures. The whole slide digital images were analyzed with HALO^®^ image analysis software version 4.0 (Indica Labs). Data were plotted and analyzed in GraphPad Prism 10.2.1 (GraphPad Software, La Jolla, CA, USA). A positive pixel count algorithm was calibrated for MPZ or LAMP1 positive staining for image analysis. The marker positivity was calculated based on the formula positivity (%) = positive area (pixels)/total analyzed area (pixels) ×100%.

### Functional testing of peripheral nerves

Evaluation of the function of peripheral nerves and stimulus transduction via the DRG was determined via the hot plate assay, which was approved by Takeda’s IACUC. The hot plate (Columbus Instruments, Columbus, OH) was preheated to 55°C. An open-ended cylindrical plexiglass tube with a diameter of 30 cm was placed on top of the hot plate to prevent mice from escaping but leaving the animal's paws exposed to the hot plate. Using a stopwatch, the time from placing the mouse on the hot plate to the time of the first paw lick were measured. Mice were removed after 1 minute if no pain response was measured to prevent tissue damage. A different set of animals from the one utilized for the MRI evaluations was used for the functional testing (n_WT _= 11; n_Fabry_+AAV_null_ = 13, n_Fabry_+AAV_Fabry_ = 13). The data were analyzed and plotted using GraphPad Prism 8.1 (GraphPad Software, San Diego, CA).

### Immunohistochemical evaluation of myelination in the paw nerves

Immunohistochemical evaluation was performed as previously reported [33].

## Results

### Test-retest repeatability of the MRI method to measure DRG CSA

**Fig 3:**
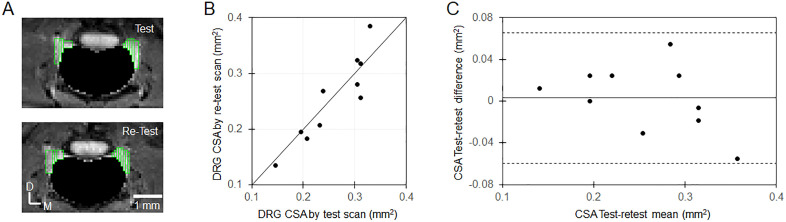
Test-retest repeatability of the MRI DRG volumetric measurement. (A) Segmented DRGs (outlined by the green triangles) from one mouse. (A) Scatterplot of the L4 DRG cross sectional area (CSA) from five mice (10 L4 DRGs) measured by test MRI scan and retest MRI scan. The bold line is the identical line. (B) Bland-Altman plot. The solid middle line denotes the bias from zero. The dashed lines show the 95% limits of agreement (i.e., bias ±1.96 × standard deviation).

To evaluate the repeatability of the MRI method to measure DRG volume, test and retest MRI data were acquired in the same five wild-type mice either during the same experiment session after re-positioning the animals (2 mice), or the retest MRI data was acquired in the next day (3 mice). S2A Fig shows the MIP images of L4 DRG from the test and retest experiments of the 5 individual mice, which look similar between test and retest imaging, and therefore qualitatively demonstrating repeatability of the DRG volumetric measurement by the MRI method. To quantitatively analyze the repeatability, DRG segmentations were performed on each DRG as shown by green rectangles in the S2B Fig. The DRG volume was measured as cross-sectional area (CSA) by the summation of all segmented pixels. The individual CSA from the test and retest MRI scans are shown in Table 2. The averaged CSA of these five wild type mice is 0.258 ± 0.063 (mean ± SD) mm^2^ by test scan, and 0.255 ± 0.076 (mean ± SD) mm^2^ by retest scan. Scatterplot and Bland–Altman plot for the Lumbar 4 DRG CSA from test and retest MRI scans are presented in Fig 3. Excellent repeatability and reliability were achieved with the limits of agreement from −0.060 to 0.066 mm^2^, and ICC of 0.9. Interestingly, the Bland–Altman plot in Fig 3B shows a trend of increasing variability with larger CSA values, suggesting that the method is slightly less precise for very large DRGs, although still within acceptable repeatability.

**Table 2:** Test-retest repeatability data from each individual mice.

Mouse	Lumbar 4 DRG	CSA test (mm^2^)	CSA retest (mm^2^)	Mean (mm^2^)	Difference	Relative difference (%)
**No.1**	Left	0.305	0.323	0.314	−0.018	−5.825
	Right	0.311	0.317	0.314	−0.006	−1.942
**No.2**	Left	0.311	0.256	0.284	0.055	19.355
	Right	0.208	0.183	0.195	0.024	12.500
**No.3**	Left	0.232	0.208	0.220	0.024	11.111
	Right	0.146	0.134	0.140	0.012	8.696
**No.4**	Left	0.330	0.385	0.357	−0.055	−15.385
	Right	0.305	0.281	0.293	0.024	8.333
**No.5**	Left	0.238	0.269	0.253	−0.031	−12.048
	Right	0.195	0.195	0.195	0.000	0.000
**Mean**		0.258	0.255	0.257	0.003	2.480
**Standard deviation**		0.063	0.076	0.068	0.032	11.340

### DRG enlargement in Fabry mice and AAV treatment effect

In the MRI study, a total of fifteen Fabry mice that were separated into 3 groups (Fabry group without any treatments, Fabry + AAV_null_ vehicle group, and Fabry + AAV_GLA_ treatment group) were evaluated between the age of week 8 to the age of week 24. Not all the 15 Fabry mice were able to survive to the end of the study. There were two Fabry mice in the AAV_null_ group which died before the age of week 24, likely due to the severity of this disease mouse model. Taguchi et al. [26] reported that the median lifespan of G3Stg/GLAko Fabry male mice is 27.6 weeks, and the death rate is ~ 25% before the age of week 24, which is consistent with the death rate in our study (2 death out of 8 mice in the two groups without the gene therapy). Interestingly, there was no death in the AAV_GLA_ group during the study.

**Fig 4:**
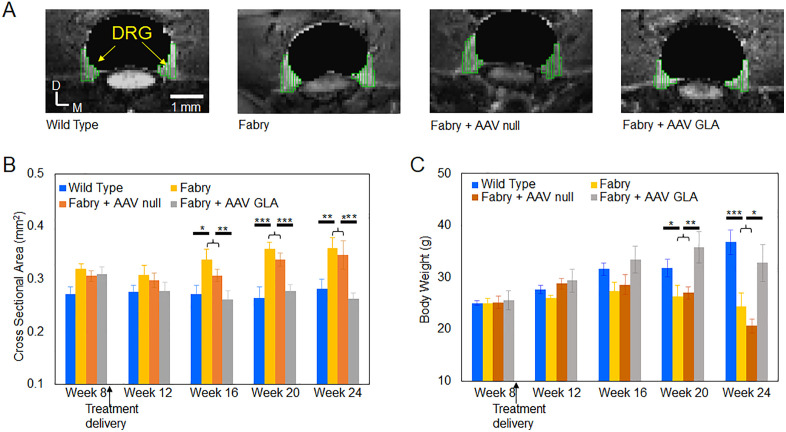
Longitudinal DRG volumetric and body weight changes in the 4 groups. (A) DRGs from one mouse at 24 weeks of age in each group were manually segmented as outlined by green rectangles. It is observable that the size of DRG in Fabry and Fabry + AAV_null_ groups is larger than in wild type and Fabry + AAV_GLA_ groups. (B & C) Longitudinal L4 DRG size and body weight in the different groups of mice (mean ± SEM). For statistical analysis, Fabry and Fabry + AAV_null_ mice were grouped together to compare with the wild type and Fabry + AAV_GLA_ mice. *: p < 0.05, **: p < 0.01, ***: p < 0.001 by student t-test.

The volumetric DRG MRI data was acquired at the ages of week 8, 12, 16, 20, and 24. S3 Fig 4 shows the DRG MRI images for each individual mouse from their last time points. The enlargement of DRGs in Fabry group and the Fabry + AAV_null_ group are observable when compared with the DRGs in the wildtype group and the Fabry + AAV_GLA_ group. As appeared in these original raw images, the size of DRGs in Fabry + AAV_GLA_ group seems to be similar with the size of DRGs in the wildtype group, suggesting that the gene therapy can normalize the DRGs in the Fabry mice.

For quantitative analysis, DRG segmentations were performed on each individual DRG as shown in Fig 4A and S4 Fig. Age-dependent DRG volume in the wild type and Fabry mice and the gene therapy effect on the DRG volume in the Fabry mice were shown in Fig 4B. The DRG volume in the Fabry mice before the start of the gene therapy at 8-week of age was higher than that of wild type mice. The DRG volume in the Fabry mice without the gene therapy (Fabry group and Fabry + AAV_null_ group) had an increasing trend from the age of 8-week to 24-week. Since the DRG volumes in the Fabry group and Fabry + AAV_null_ group were not statistically different, they were pooled together for quantitative comparison with the wildtype group. From the age of 16-week and after, the DRG sizes in the Fabry group and Fabry + AAV_null_ group were larger and statistically different than the DRG sizes in the wildtype group. At the age of 24-week, the DRG size in the Fabry mice was ~ 25% larger and statistically different than the DRG size in the wild type mice (CSA of 0.35 mm^2^ for the Fabry mice vs. CSA of 0.28 mm^2^ for the wild type mice).

To detect the gene therapy effect, the DRG size in the Fabry + AAV_GLA_ group was compared with the DRG size in the wildtype group and the two Fabry groups without the gene therapy. There were no statistically significant differences at all the time points between the gene therapy group and the wildtype group. However, eight weeks after delivering the gene therapy (i.e., at the age of 16-week), the DRG volume in the gene therapy group was significantly smaller than the DRG volume in the Fabry mice without the gene therapy and the effect was sustained even until week 24 (Fig 4B). Our results suggest that gene therapy normalized the DRG volume in the Fabry mice.

Fig 4C shows the body weights changes from the age of 8-week to 24-week. The body weights in the wildtype group and the gene therapy group had an increasing trend from the age of 8-week to 24-week. Like the DRG volumes in the Fabry group and Fabry + AAV_null_ group, the body weights in these two groups were not significantly different, and they were pooled together for quantitative comparison with the wildtype group and the gene therapy group. The body weights of the Fabry mice without the gene therapy were significantly lower than the wild type group and the gene therapy group starting at the age of 20-week, while the body weight in the gene therapy was similar to the body weight in the wild type group at all time points, indicating that AAV-based gene therapy could normalize the body weight in the Fabry mice. A previous report from human study showed that the DRG volume correlated with body weight in healthy adults aged between 23–79 years (see Fig 4 in [34]), Therefore, our data in the Fabry mice that received gene therapy and showed that their DRG size did not increase with the body weight gain suggest that the normalization of DRG size was likely attributed to the effect of gene therapy, and plausible this may have been attributed to lower Gb3 accumulation in the DRG.

### DRG tissue histological analysis

**Fig 5:**
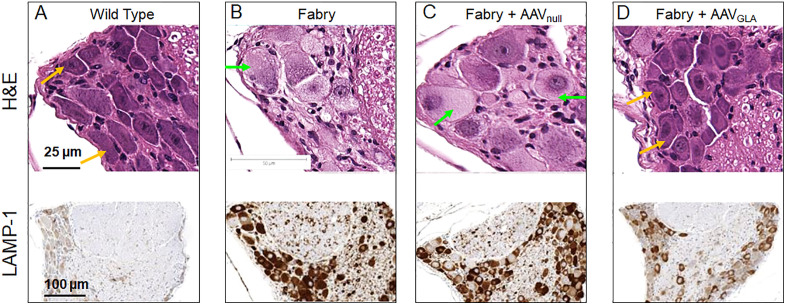
Histopathological evaluation of the dorsal root ganglion. Tissues were collected from mice at the end of the MRI study at 24-weeks of age. Hematoxylin and eosin (H&E) staining (top row) shows normal neurons in wild type mouse (yellow arrows in **A)**, vacuolation in neurons of the DRG in the Fabry mouse (B) and the Fabry mouse with the vehicle treatment (green arrows in B & **C)**, and normal neurons in the Fabry mouse with the gene therapy (yellow arrows in **D)**. The vacuolated and enlarged DRG neurons in the 24-week-old Fabry mice are evident when compared to the wildtype mouse and the Fabry mouse with gene therapy, especially in the mouse with vehicle treatment. LAMP-1 staining (bottom row) shows increased LAMP-1 protein levels in the Fabry mouse without the gene therapy (B) compared to the wildtype mouse (A) and the Fabry mouse with the gene therapy **(C)**.

Considering that in Fabry disease, the enlargement and vacuolation of cells manifest as organ enlargement, histological analysis was performed to confirm the MRI finding of the DRG enlargement in the Fabry mice. H&E-stained cross sections of the L4 DRG revealed hypertrophied DRG neurons with vacuolated cytoplasm in Fabry mice (Fig 5B). H&E images from the age-matched control wild type mice were unremarkable, and DRG neurons had a uniform, granular basophilic cytoplasm (Fig 5A). The histology analysis results confirmed the pathological changes in DRG neurons of the Fabry mice and supports volumetric DRG MRI as a non-invasive imaging biomarker to monitor pathological changes in the DRG neurons of Fabry mice.

### Effect of AAV treatment on the lysosomal burden of the DRG

The major histopathological hallmark in Fabry disease is the elevated lysosomal burden, due to accumulation of Gb3 within this organelle. Along with increased lysosomal biogenesis to accommodate the storage of Gb3 and support of other catabolic processes, samples from Fabry patients and animals exhibit high numbers of lysosomes, determined through the detection of lysosome-specific markers, like LAMP1 (Lysosomal-associated membrane protein 1). After completion of the MRI evaluation, we obtained spinal cord tissues and assessed the lysosomal burden in the DRG using LAMP1-based immunohistochemistry. Our results indicated that G3Stg/GLAko Fabry mice treated with AAV_GLA_ had marked reduction in LAMP1 staining, contrary to AAV-treated mice that had extensive and strong LAMP1 staining (Figs 5C and 5D). These data corroborate the MRI-based observation that reduction in DRG volume after AAV_GLA_ is attributed to the lowering of lysosomal burden due to concomitant reduction of Gb3 levels within these cells.

### Effect of AAV treatment on peripheral nerve sensation

**Fig 6:**
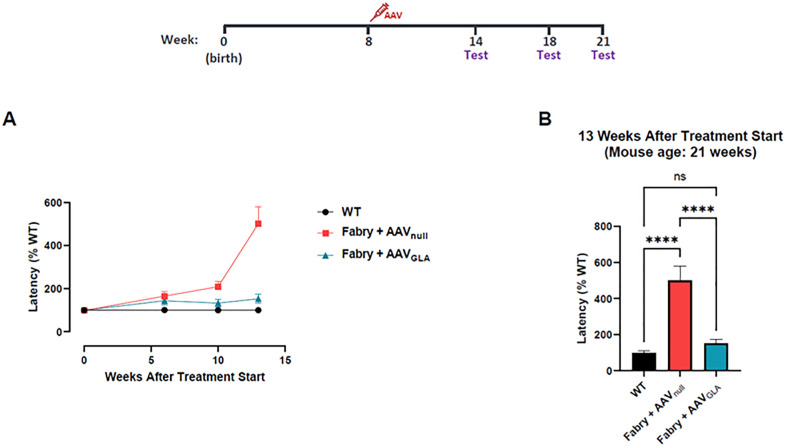
Gene therapy restores peripheral nerve sensation in the paw of Fabry (Gb3STg/GLAko) mice. (A) Longitudinal functional testing via the hot plate assay in wild type (WT), AAV_null_-treated and AAV_GLA_-treated Fabry mice (mean ± SEM). (B) Comparison of the latency at 13 weeks post treatment initiation, demonstrating restoration of sensation in the AAV_GLA_-treated animals (mean ± SEM, ns: not significant, ****: p < 0.0001 based on one-way ANOVA).

Due to accumulation of Gb3 in the DRG, neuronal inflammation and loss of myelinated and nonmyelinated neuronal fibers, Fabry patients experience peripheral neuropathy, including neuropathic pain, and reduced cold or warm sensation^7^. Towards this, we examined whether gene therapy with AAV_GLA_ could improve peripheral sensory function in G3Stg/GLAko Fabry mice, using the hot plate assay. The latency to respond in AAV_GLA_-treated mice was similar to normal controls 6 weeks after treatment administration and was not statistically different at the end of the study, indicating preservation of sensory function (Fig 6). In contrast, AAV_null_-treated showed progressive impairment of peripheral nerve sensation, reflected in increased latency in the heat plate test (Fig 6A). At the age of 21 weeks and after 13 weeks of gene therapy treatment, the response to heat sensation was normalized to the WT control, while the AAV_null_ group had significantly increased latency, which was up to 5 folds from the WT or baseline (Fig 6B). This demonstrated the prevention of thermal sensation deficit in AAV_GLA_-treated G3Stg/GLAko Fabry mice and shows that gene therapy could provide phenotypic and functional peripheral nerve improvements in Fabry disease, as determined through MRI and hot plate assay respectively.

### Myelination

**Fig 7:**
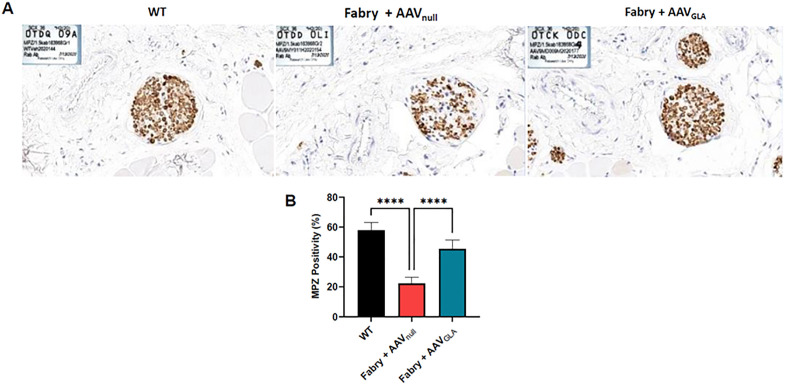
Immunohistochemical evaluation showing increased myelination in the paw nerves of Fabry (Gb3STg/GLAko) mice after gene therapy. (A) Representative images of paw nerves from wild type (WT), AAV_null_-treated and AAV_GLA_-treated Fabry mice stained for Myelin protein zero (MPZ, brown color), which is a major component of the myelin sheath in the peripheral nervous system. (B) Quantification of the MPZ immunohistochemistry data (mean ± SEM; n_WT_ = 11; n_Fabry+AAVnull_ = 12; n_Fabry+AAVFabry_ = 13; ****: p < 0.0001 based on one-way ANOVA).

To further confirm the MRI phenotypic and functional observations, we used immunohistochemistry, for molecular characterization of the DRG. Towards this, the myelination of the paw nerves of wildtype and Fabry mice was evaluated, since a hallmark of the Fabry disease is a decrease in the density of small unmyelinated and thinly myelinated dermal nerve fibers. Staining for myelin protein zero (MPZ) revealed that there was loss of myelinated fibers in the paws of the control Fabry mice, but the paws of GT-receiving Fabry mice had more myelinated fibers, which were comparable to those of wildtype animals. These data support the hypothesis that GT directly exerts a therapeutic effect at the site of the pathology, which can be evaluated with MRI and confirmed *ex vivo* Fig 7.

## Discussion

### Summary of findings

The present work demonstrates that MRI can be used for the *in vivo* monitoring of response to treatment as part of drug development process, which can be used in animal models with DRG manifestations, including Fabry disease, Sjögren disease, Friedreich’s Ataxia and other sensory neuropathies. The major findings of this study are: (1) a MRI protocol with Bruker’s fast spin-echo RARE sequence was developed to measure DRG volume non-invasively, *in vivo* in both wildtype and Fabry mice; (2) a new volumetric DRG MRI method was established that has a high reproducibility with an intraclass correlation coefficient of 0.9; (3) there is no change in DRG size in C57BL/6 wild-type male mice after the age of 8 weeks; (4) enlargement of DRG in the Fabry mice can be reliably observed by the MRI method; and (5) histological analysis of the DRG tissue from the Fabry mice shows enlarged and vacuolated DRG neurons, confirming that this MRI method has the sensitivity to measure pathologically-induced volume change in DRG.

### MRI measurement of DRG size in mice

In this study, mouse DRG volume was successfully measured *in vivo* by high resolution T2-weighted MRI. Since this is the first report of MRI observation of DRG in live mice, MRI data was carefully evaluated on an animal-by-animal basis to ensure that the observed DRG measurements are reliable. As demonstrated in Fig 3, 4, and 5, the volumetric DRG MRI method presented in this report has high reproducibility and can measure pathology-induced DRG volume changes in Fabry mice. Several factors contributed to the success of this study. First, the use of a small 10-mm surface coil was a key factor. Due to the small size of mouse DRG, an MRI method with a high spatial resolution is needed which requires high sensitivity to acquire MRI images with an acceptable signal-to-noise ratio. The coil is a key factor in achieving such high sensitivity. Since we focused only on the L4 DRG in this study, a small 10-mm surface coil which is located closely to the L4 segment of spinal cord was selected as the receiver coil. Using a small surface coil for high resolution MRI has been used before to study the sub-millimeter columnar structure in the visual cortex of cats [35,36], and micro brain structures in mice [37]. Second, to minimize the impacts of respiratory motion on MRI images, the surface coil was fixed on the animal bed, and the mouse was positioned on the coil in the supine position. The small surface coil is only sensitive to the limited volume surrounding the L4 segment of DRG, and tissues from the chest and upper-abdominal regions have limited contributions to the MRI signal, which helps to minimize the image artifacts caused by respiratory motion. Third, the animal is carefully positioned with its spinal cord to be parallel with the z-direction of the magnet. As shown in Supplementary Fig. 1, the adjacent spinal cord without the DRG is used to delineate the DRG from the spinal cord. If the spinal cord is not parallel to the z-direction of magnet, it would be tilted in the lateral-lateral direction. The spinal cord in the adjacent spinal cord-only slices would not be co-localized with the spinal cord in the slices with DRG and the delineation of DRG from the spinal cord would not be easily performed. Even though manual shifting can be used to correct this mis-registration, it would add another variable that could impact the final DRG volume quantification.

One interesting finding of this study is that the location of DRG is different from what has been observed in humans and other animal species. In humans, most DRGs are located in foraminal position [24], which are connected with spinal cord via the dorsal nerve root [7,15,38,39]. In NHPs, dogs, and rats, the DRGs are partially attached to spinal cord [18,40]. In mice, however, the whole DRG are closely attached with spinal cord as shown in Fig 2. This close attachment of DRG to spinal cord in mice has been reported by direct observation of the excised spinal column (see Fig 1I in [2]). In the dorsal-ventral direction, the DRG in mice are visible in the lateral-ventral side of spinal cord as shown in Fig. 2, not the lateral-dorsal side of spinal cord, which is consistent with what has been reported in rats (see Fig 5 in [20]).

### DRG enlargement in Fabry mice

In this study, volumetric DRG MRI shows enlargement of DRG in Fabry mice (Fig 4 & 5), which is consistent with the observation from a recent DRG MRI study in mice [41]. Our histological analysis of DRG tissue shows hypertrophied and vacuolated DRG neurons (Fig 6), suggesting that the macroscopically enlarged DRG observed in our study is caused by the hypertrophic/vacuolated DRG neurons. The observation of hypertrophic/vacuolated DRG neurons has been reported before in both human Fabry patients [42], and Fabry mice [43–46], but the causal relation between hypertrophic DRG neurons and DRG volume has not yet been confirmed in those studies. However, in studies investigating cardiac hypertrophy in human Fabry patients, the causal relation between hypertrophic/vacuolated cardiomyocytes and cardiac hypertrophy has been established [47–50], suggesting that the enlarged DRG observed by MRI in our study is caused by the hypertrophic and vacuolated DRG neurons. Since observed vacuolization in cells is caused by lysosomal Gb3 accumulation [5], in-life MRI measurement of DRG enlargement in Fabry mouse can be used as a biomarker for the Gb3 accumulation in DRG neurons, evaluating the disease progression and an efficacy for Gb3 clearance by a therapeutic approach. This non-invasive biomarker would be a significant and powerful method for Fabry disease drug discovery efforts or any other indications which need to monitor DRG size.

In addition to enlarged/vacuolated DRG neurons, local inflammation in DRG may also contribute to the DRG enlargement. Choconta *et al* reports that the increased macrophage CD68 expression indicative of enhanced phagocytic activity can be observed in DRG of Fabry mice [46], indicating the inflammatory processes within DRG, potentially leading to increasing DRG volume through local edema. Since MRI T2 signals represent a reliable surrogate marker for edematous/inflammatory processes in tissues [51], MRI studies focusing on T2 measurement can be performed to evaluate the potential contribution from the inflammation to the observed DRG enlargement in Fabry mice. Significantly, recent publications have demonstrated the value of MRI T2 measurement in DRG with Fabry patients [12,52], but T2 measurement in mouse DRG has not been reported previously. The DRG size observed from this study suggests that it is possible to perform T2 MRI in mouse DRG. As shown in Fig 2, the DRG size in rostral-caudal direction is about 0.9 mm, which means that MRI data from a single axial slice with a slice thickness of ~0.5 mm centered within the DRG can be acquired. The DRG size in the axial slice can allow the in-plane resolution of ~0.15 mm to adequately observe DRG. Such 0.15 × 0.15 × 0.5 mm^3^ spatial resolution may have enough signal-to-noise ratio for a reliable T2 measurement. Furthermore, Cryo-coil which can increase SNR by 2–2.5 times comparing with regular surface coil can further boost the feasibility for DRG T2 measurement in mice [53].

### AAV treatment effect on the DRG volume in the Fabry mice

A major limitation of current therapies for Fabry disease is their limited tissue exposure and retention, especially for tissues like the heart and nerves. This may be attributed to the fact that these therapies use the native α-GAL protein, which is an intracellular enzyme with low stability in circulation. The observations of the current study that treatment with AAV encoding α-GAL ameliorates the disease burden at the DRG of Fabry mice aligns with a recent report that showed uniform clearance of Gb3 in DRG neurons [33], as opposed to earlier results that showed that some neurons were refractory to treatment [28]. Our findings that GT could confer phenotypic and functional DRG improvement in Fabry mouse models provide a potential therapeutic advancement for individuals with this rare disease.

### Volumetric MRI as a translational biomarker for gene therapy in Fabry disease

The peripheral nervous system is affected in patients with Fabry disease, including accumulation of Gb3 in DRG that results in sensory deficiency and small fiber neuropathy. Although DRG stimulation is used in clinics to manage chronic nerve pain, the longitudinal non-invasive evaluation of disease progression in DRG is challenging. Considering MRI’s broad presence in the clinical setting and the data presented herein, volumetric MRI provides a unique translational opportunity to perform natural history studies and determine the therapeutic effect of emerging therapies in Fabry patients. It also allows the collection of DRG patient data to further characterize peripheral neuropathy in Fabry patients, since molecular and phenotypic characterization of peripheral neuropathy manifestations in these patients have been limited [3,54].

### Limitations of our MRI method and future directions

Several limitations of the present study should be addressed, along with potential improvements for future research. First, in this study, we used the cross-sectional area (CSA) derived from maximum intensity projection (MIP) images in the axial direction as a surrogate for dorsal root ganglion (DRG) volume. For more precise CSA quantification, thinner slices would be preferable. Thin slices can minimize partial volume effects in boundary voxels, leading to sharper and more accurate boundaries. In our study, the slice thickness was limited to 0.292 mm, which, when compared to the submillimeter size of the DRG, may lead to blurred boundaries due to partial volume effect. To improve spatial resolution with thinner slices, we suggest exploring the use of a Cryo-coil, which could enhance sensitivity. Studies have shown that the signal-to-noise ratio (SNR) of a Cryo-coil, compared to a surface coil of similar geometry, can increase by 2–2.5 times [53]. With such an improvement of SNR, slice thickness could potentially be reduced to 0.12–0.15 mm, thereby minimizing partial volume effects and improving the accuracy of CSA measurements, which may even enable direct volumetric measurement through DRG segmentation on slice-by-slice basis.

Secondly, we used the adjacent spinal cord images for DRG delineation, as no clear contrast between the DRG and spinal cord was observed (Fig 2). The reason for the lack of clear contrast between the DRG and spinal cord in our T2-weighted data is not immediately apparent. However, based on data from recent studies, we know that the T2 values of the DRG and spinal cord are distinct. For instance, Schindehütte et. al, reports that the T2 of the DRG at 3T is approximately 40 ms [12], while Stanisz et. al, found that the T2 of the spinal cord at 3T is around 78 ms [55]. We would expect a similar trend at 7T, meaning the DRG should have a lower signal intensity compared to the spinal cord in the T2-weighted images. The absence of clear contrast in our data could partly be due to the relatively short effective echo time (TE) used in our study (42 ms). Increasing the TE may improve the contrast between the DRG and spinal cord, and could be an avenue for future exploration. Another potential method to enhance contrast is diffusion MRI. The spinal cord consists of gray matter at the center, surrounded by white matter which is composed of longitudinal axons. If a diffusion gradient is applied in the z-direction (parallel to the white matter axons), the signal in the outer layers of the spinal cord, adjacent to the DRG, would be more significantly suppressed. This would enhance the contrast between the DRG and the outer layer of the spinal cord, potentially making it easier to directly delineate the DRGs.

Thirdly, the high-intensity signals observed outside the spinal cord were regarded as originating from the DRG in this study. Although the high-intensity regions may include contributions from nerve roots, blood vessels, or partial volumes of CSF, the DRG should represent the major component. As shown in Fig 4, differences in the measured DRG size were observed between Fabry mice and wild-type mice, and the longitudinal changes in DRG size also differed between the two groups. These differences are likely attributable primarily to changes in DRG size, since contributions from minor components are expected to be similar between Fabry and wild-type mice. More importantly, the therapeutic effect of AAV on DRG size in Fabry mice was detectable using our MRI method, further supporting that the high-intensity regions outside the spinal cord mainly reflect DRG.

## Conclusions

We demonstrate that MRI can measure pathology-induced volumetric changes in DRG of Fabry mice *in vivo* and the normalization of DRG volume after AAV-based gene therapy. Volumetric DRG MRI can be a valuable tool for medical research and for developing translational biomarkers to support drug discovery.

## Supporting information

S1 File Consistency in the shape and size of spinal cord within the five consecutive 0.29 mm slices. The spinal cord in Slice No.1 was outlined with the green contour, this green contour was then overlaid on all other slices.
(PDF)
