# Supplementary material for: Volumetric MRI of dorsal root ganglia as a biomarker for disease progression and response to AAV treatment in a mouse model of Fabry disease
Source: PLoS One. 2025 Oct 24;20(10):e0334840. doi: 10.1371/journal.pone.0334840 (PMC12551818; doi:10.1371/journal.pone.0334840)
Supplement: S1 File — The spinal cord in Slice No.1 was outlined with the green contour, this green contour was then overlaid on all other slices. (PDF) [file pone.0334840.s001.pdf]

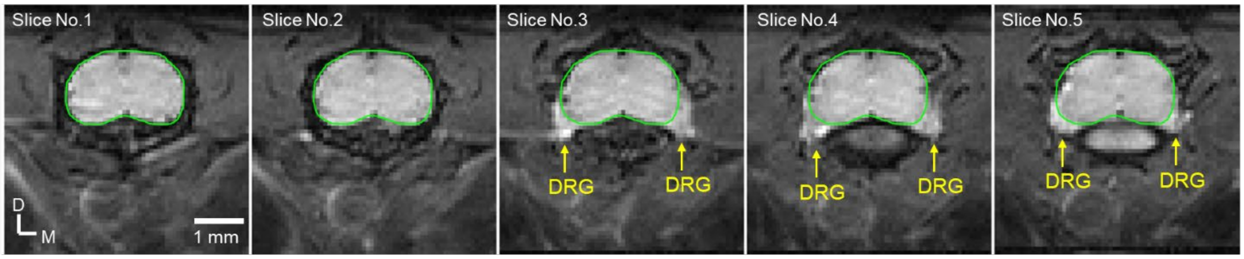

S1 Fig. Consistency in the shape and size of spinal cord within the five consecutive 0.29 mm slices. The spinal cord in Slice No.1 was outlined with the green contour, this green contour was then overlaid on all other slices.

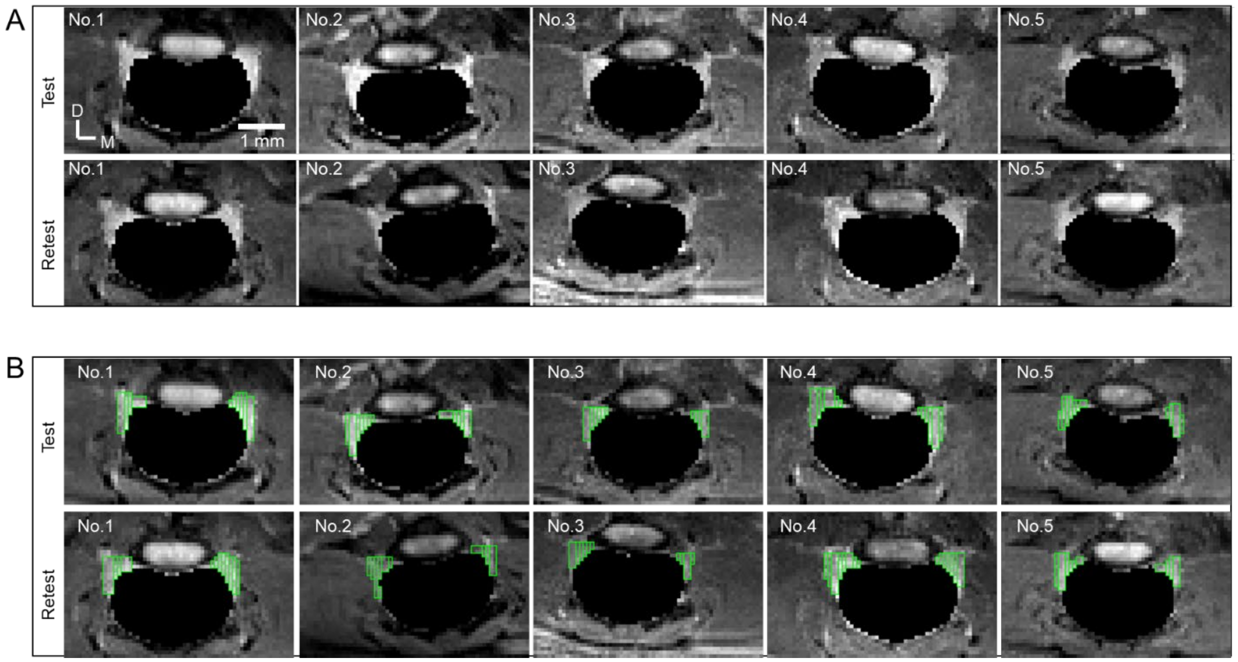

S2 Fig. (A) DRG observation in each individual mouse from the test-retest study. (B) DRGs were manually segmented as outlined by green rectangles. D: dorsal, M: medial.

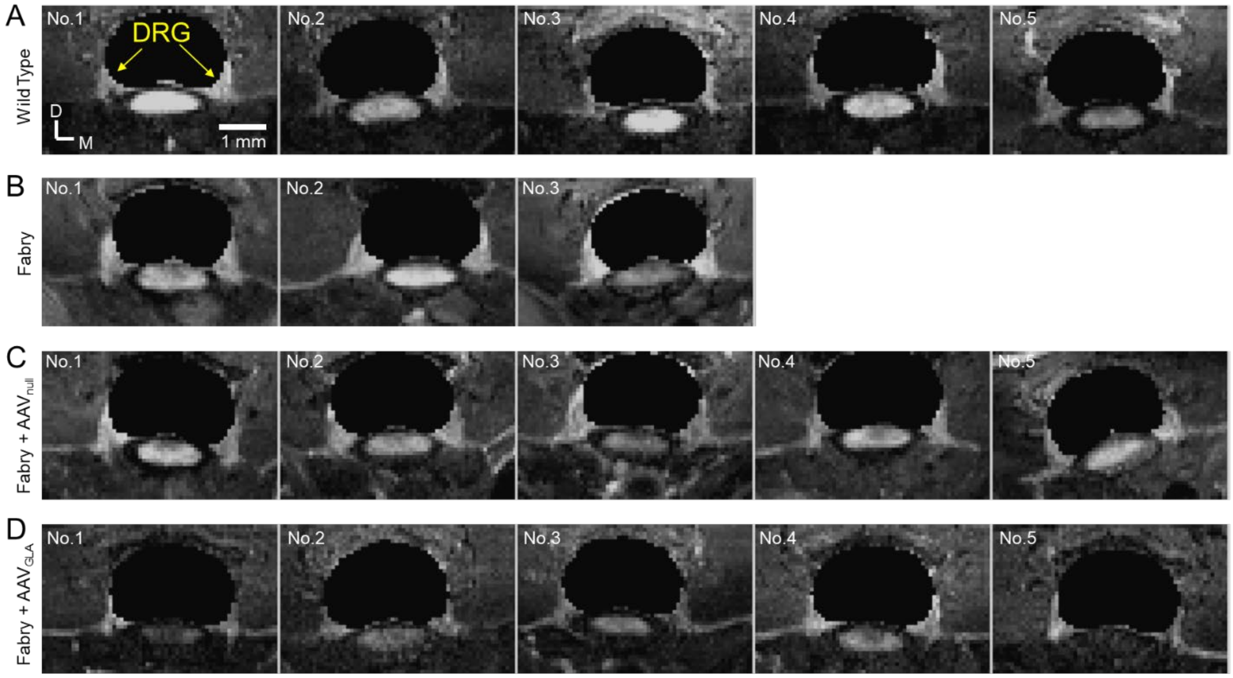

S3 Fig. L4 DRG of each individual mice in the wildtype group (A), Fabry without any treatment group (B), Fabry with the vehicle treatment (AAV_{null}) group (C), and Fabry with the gene therapy (AAV_{GLA}) group (D). The DRG enlargement in Fabry mice without gene therapy compared to wildtype mice and Fabry mice with gene therapy is observable. D: dorsal, M: medial.

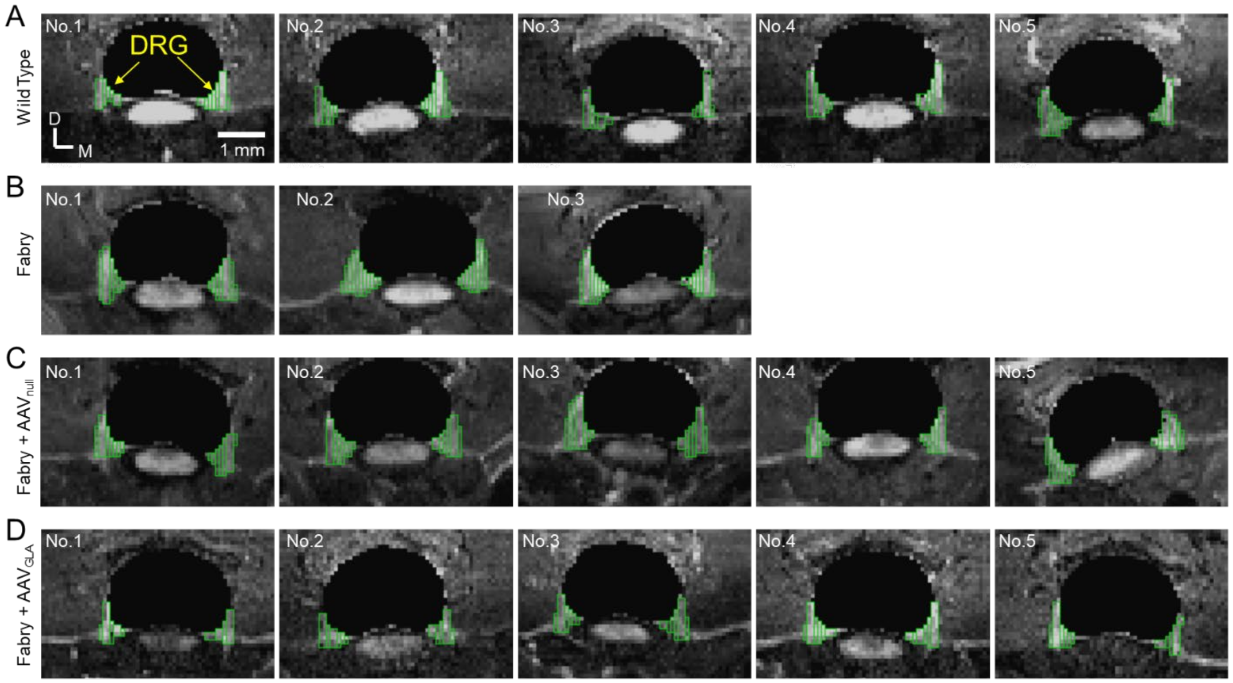

S4 Fig. Segmented L4 DRGs of each individual mice in the wildtype group (A), Fabry without any treatment group (B), Fabry with AAV_{null}-treatment group (C), and Fabry with the gene therapy (AAV_{GLA}) group (D). The DRG enlargement in Fabry mice without gene therapy compared to wildtype mice and Fabry mice with gene therapy is observable. D: dorsal, M: medial.

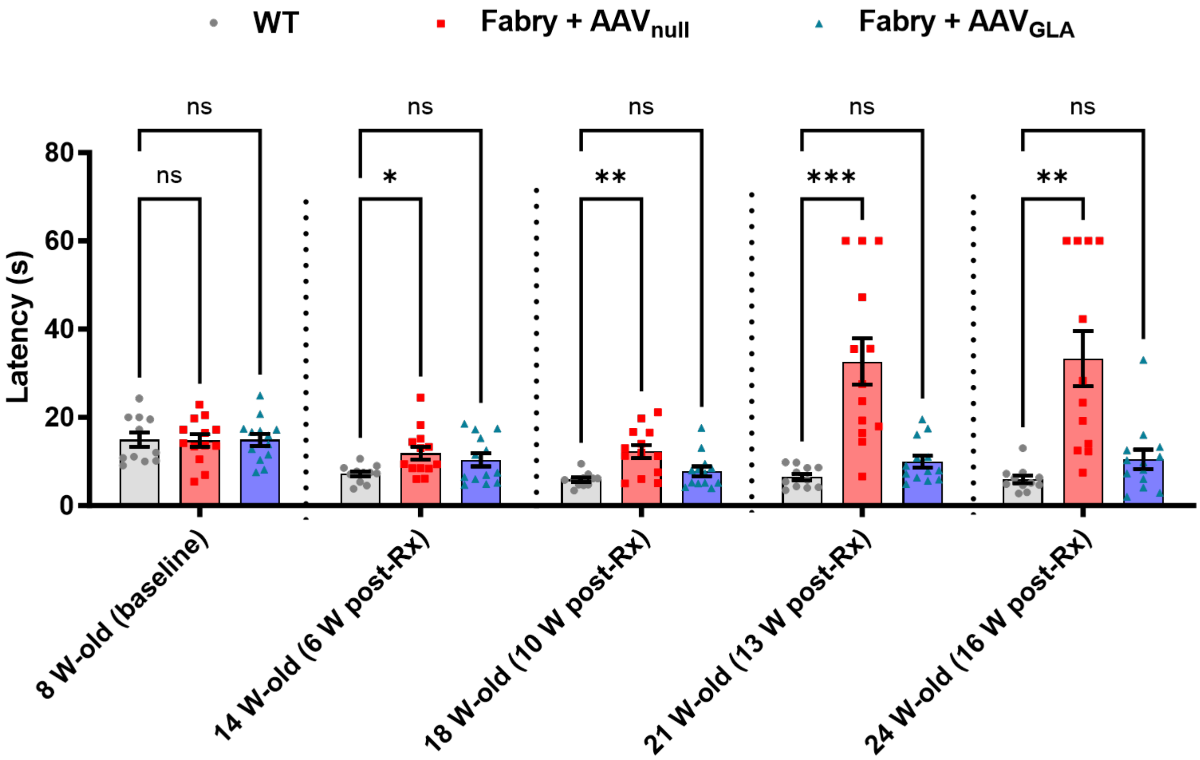

S5 Fig. Gene therapy restores peripheral nerve sensation in the paw of Fabry (Gb3STg/GLAko) Mice. Longitudinal functional testing via the hot plate assay in wildtype (WT), $AAV_{null\text{-}treated}$ and $AAV_{GLA\text{-}treated}$ Fabry mice (mean ± SEM; $n_{WT} = 11$; $n_{Fabry+AAVnull} = n_{Fabry+AAVFabry} = 13$; ns: not significant, *: $p < 0.05$, **: $p < 0.01$, ***: $p < 0.001$ based on two-way ANOVA).
